# Echocardiographic monitoring of myocardial function in a female patient with right heart Loeffler endocarditis at thrombotic stage after Epstein-Barr-virus infection

**DOI:** 10.1007/s10554-024-03147-2

**Published:** 2024-05-23

**Authors:** Joscha Kandels, J Pawluczuk, Stephan Stöbe, Andreas Hagendorff

**Affiliations:** Klinik und Poliklinik für Kardiologie, Universitätsklinikum Leipzig, Liebigstr. 20, Leipzig, 04103 Germany

**Keywords:** Thrombotic loeffler endocarditis, 3D echocardiography, Deformation imaging, Speckle tracking, Disease monitoring

## Abstract

**Background:**

Transthoracic echocardiography is usually the first non-invasive imaging modality for the detection of Loeffler endocarditis at thrombotic stage. In the recent decade 3D echocardiography and deformation imaging already proved as a helpful tool for the monitoring of left and right ventricular heart disease.

**Case presentation:**

The present case illustrates the diagnostic role of 3D echocardiography and deformation imaging in the acute stage of right sided Loeffler endocarditis in a 70-year-old Western European (German) woman. This case proves that myocardial involvement due to inflammation can be detected at subclinical stages by speckle tracking echocardiography. Acute deterioration of left and right ventricular function and the early response to prednisolone therapy can objectively be monitored. In addition, alterations of effective stroke volume can quantitatively be assessed by 3D right ventricular volumetry with exclusion of thrombus formation in the volume measurements.

**Conclusion:**

This case underlines the importance of 3D echocardiography and deformation imaging as a helpful diagnostic tool in disease management in the acute phase of Loeffler endocarditis at thrombotic stage.

**Supplementary Information:**

The online version contains supplementary material available at 10.1007/s10554-024-03147-2.

## Background

Loeffler endocarditis is the result of eosinophilic infiltration of the myocardium [1–3]. The possible cause can be a hypereosinophilic reaction due to drugs or parasites [4]. Also isolated tissue infiltrations without peripheral hypereosenophilia are described in literature [1, 5]. Viral infection might cause hypereosinophilic syndrome (HES) resulting in clonal expansion of T2 helper lymphocyte and exertion of cytokines promoting hypereosinophilia [6]. The cause of HES might also be idiopathic [5].

Loeffler endocarditis is associated with pathomorphological changes in the endomyocardium starting with acute myocardial inflammation, followed by isolated subendocardial muscle necrosis due to eosinophilia with myotoxic deposits of eosinophils, fibrin and thrombotic material [2, 7, 8]. The end stage of chronic endomyocardial fibrosis is characterized by myocardial remodeling and restrictive cardiomyopathy [3, 9, 10].

Prognosis depends on the severity of the acute myocardial eosinophilic infiltration, the subsequent myocardial fibrosis and the arrhythmogenic and thromboembolic complications. The arrhythmogenic substrate of malignant arrhythmias is related to the severity of myocardial edema and tissue necrosis, which is generally accompanied by severe impairment of myocardial function. Fulminant course of Loeffler endocarditis is most often described in the acute phase [1, 11, 12]. A special form of Loeffler endocarditis is the isolated right ventricular (RV) involvement [3, 9, 10, 12, 13] as presented in this case.

The risk of acute heart failure, malignant arrhythmias and peripheral thrombotic at the early stage of Loeffler endocarditis underlines the importance of subtle diagnosis of myocardial involvement. Thus, modern imaging techniques – especially non-invasive echocardiography – might play a central role in the management of those patients. In addition, successful treatment could be demonstrated by early follow-up investigation documenting significant improvement of left ventricular (LV) and RV function by deformation imaging. The assessment of effective cardiac stroke volume, cardiac output and cardiac index underlines the importance of quantitative echocardiography in hemodynamic monitoring of those patients.

## Case Presentation

**Fig. 1:**
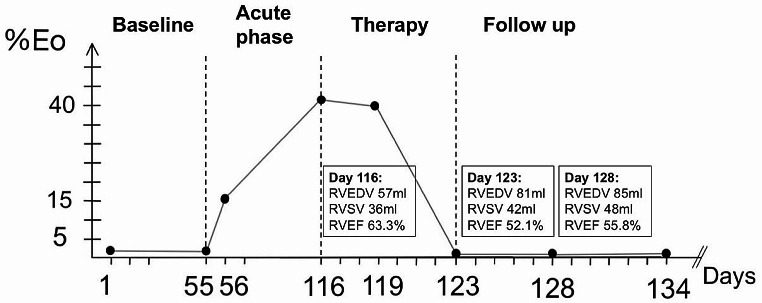
Time course of percentage eosinophils (%Eo): Changes of Eo% from baseline to follow-up at day 134. At day 55 patient was admitted to the emergency room (ER) with the described reactivation of Epstein-Barr-Virus infection (EBV). Day 116 precedes Loeffler diagnosis. Day 119 precedes prednisone therapy by 24 h. Day 123 follows beginning of prednisone therapy. Day 134 was laboratory control at follow-up. RVEDV: right ventricular end diastolic volume; RVSV: right ventricular stroke volume; RVEF: right ventricular ejection fraction

The actual disease history of a 70-year-old female patient started with a hospital admission due to syncope in the presence of paroxysmal atrial fibrillation. Baseline conventional echocardiography showed no abnormalities – especially of the RV. Discharge occurred with metoprolol and apixaban therapy. Two months later, she was admitted to the emergency room with clinical signs of increasing fatigue, reduced performance, and dyspnea on exertion. She reported a recent weight loss of 3 kg with subfebrile temperatures for previous 2–3 weeks. The patient had the oncological history of squamous cell carcinoma of the cervix, at TNM stage pT1b1 pN0 (0/15) M0 L1 V0 Pn0 R0 G2. Three years ago, the patient underwent a total mesometrial resection of the uterus, pelvic first line lymphonodectomy and ovariectomy. Diagnostic evaluations revealed the presence of a hypereosinophilia (15%), hemorrhagic pleural effusions, pericardial effusion, and a reactivation of an Epstein-Barr virus (EBV) based on polymerase chain reaction testing from pericardial punctuate. Due to pericarditis patient received colchicine therapy. Two weeks later, she was admitted with severe hypereosinophilia (42%) (Fig. 1). Echocardiography showed global hypokinesia of the mid apical LV myocardium causing moderately reduced systolic function, elevated E/E´-ratio and a significant thrombus formation in the RV apical region, documenting a thrombotic stage of Loeffler endocarditis of the RV, and no pericardial effusion. Regional myocardial tissue characterization was performed by cardiac magnetic resonance tomography (CMR) showing acute inflammation and edema as well as diffuse late gadolinium enhancement in the RV free wall as well as in the mid apical septal regions of the LV. Prednisolone therapy was initiated inducing a rapid decrease of eosinophils to normal values within two days. Echocardiographic follow-ups were performed to monitor LV and RV function and to characterize the impact on cardiac hemodynamics.

**Fig. 2:**
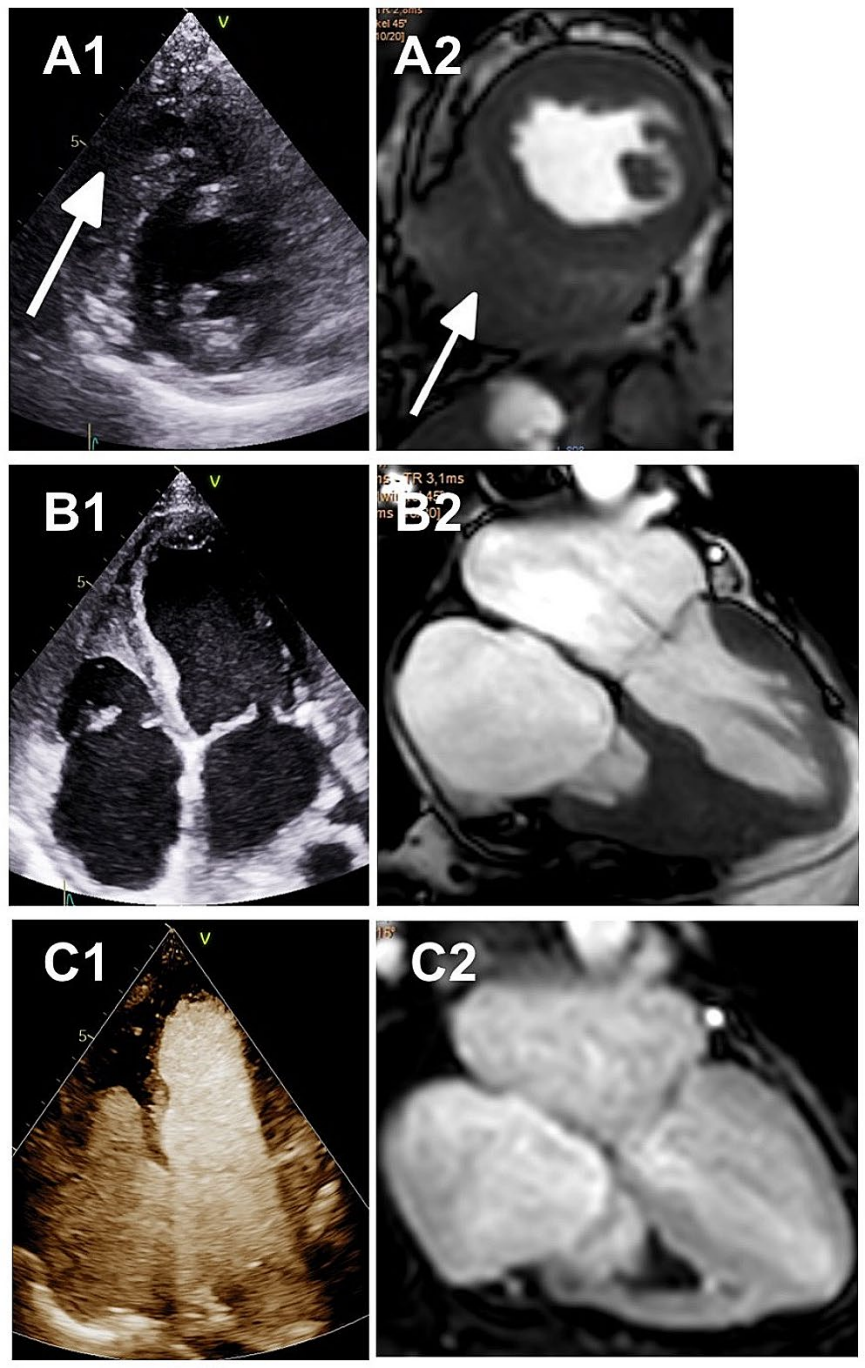
Images from transthoracic echocardiography (TTE) – native 2D images and contrast echocardiography (1), and cardiac magnetic resonance tomography CMR – cine T1 and perfusion sequences (2) prior to prednisone therapy: (**A**) Parasternal midapical short axis view with the thrombus formation (arrow) in the right ventricular (RV) apical region. (**B**) 4 chamber view illustrates thrombus formation in the midapical RV cavity. (**C**) Contrast echocardiography and perfusion sequences by CMR also illustrate the RV mass as a thrombus formation

The main finding of the RV thrombus formation by transthoracic echocardiography (TTE) and CMR is displayed in Fig. 2.

**Fig. 3:**
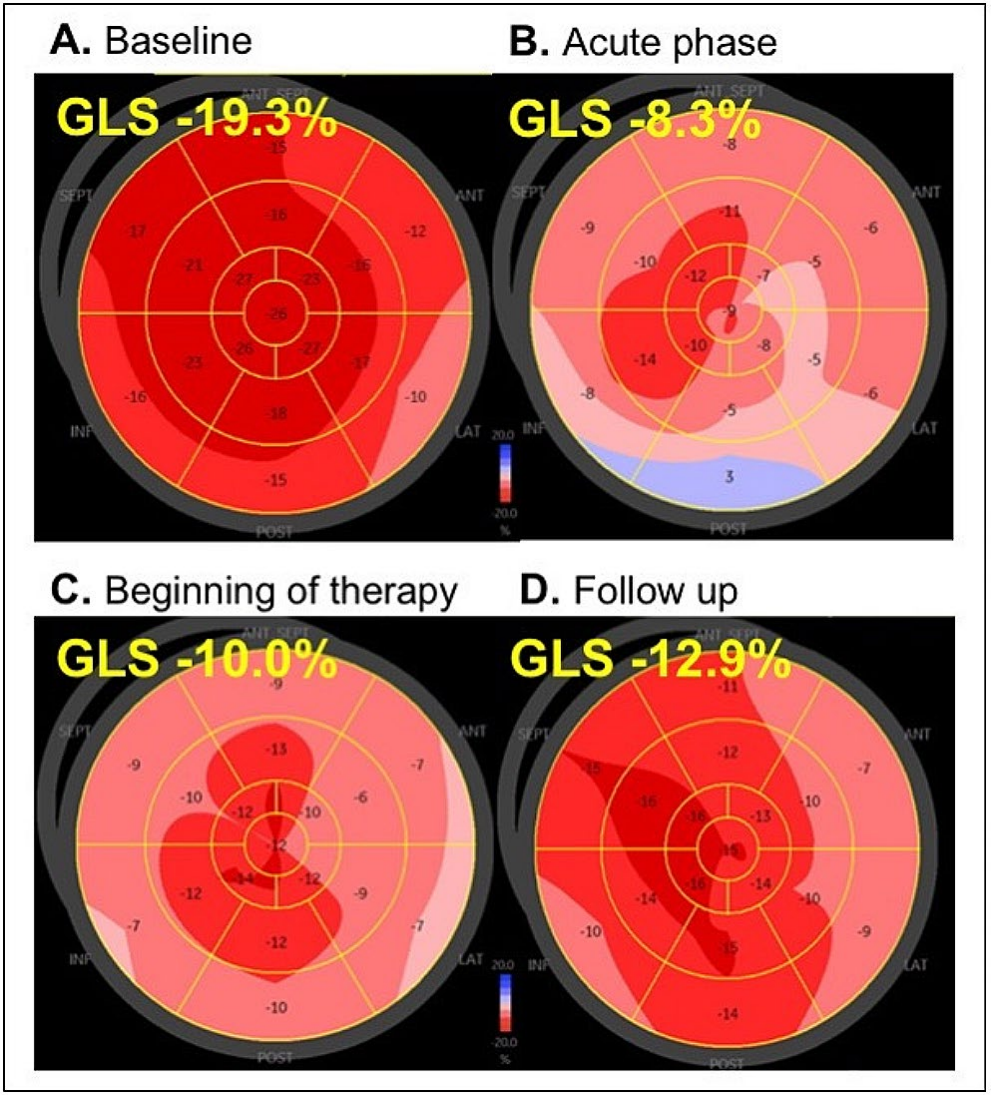
Patterns of left ventricular longitudinal strain at baseline (**A**), acute stage of inflammation (**B**), at early prednisolone therapy (**C**) as well as two weeks under ongoing immunosuppressive therapy (**D**): At baseline, no ventricular wall motion abnormalities were found (A – GLS = -19.3%). In the acute phase almost 3 months later average global peak systolic strain value significantly increased (B – GLS = -8.3%). There has been an improvement in wall motion at the follow up to prednisone therapy (C – GLS = -10%). Left ventricular motion further improved by the time in the follow up (D – GLS = -12.9%)

**Fig. 4:**
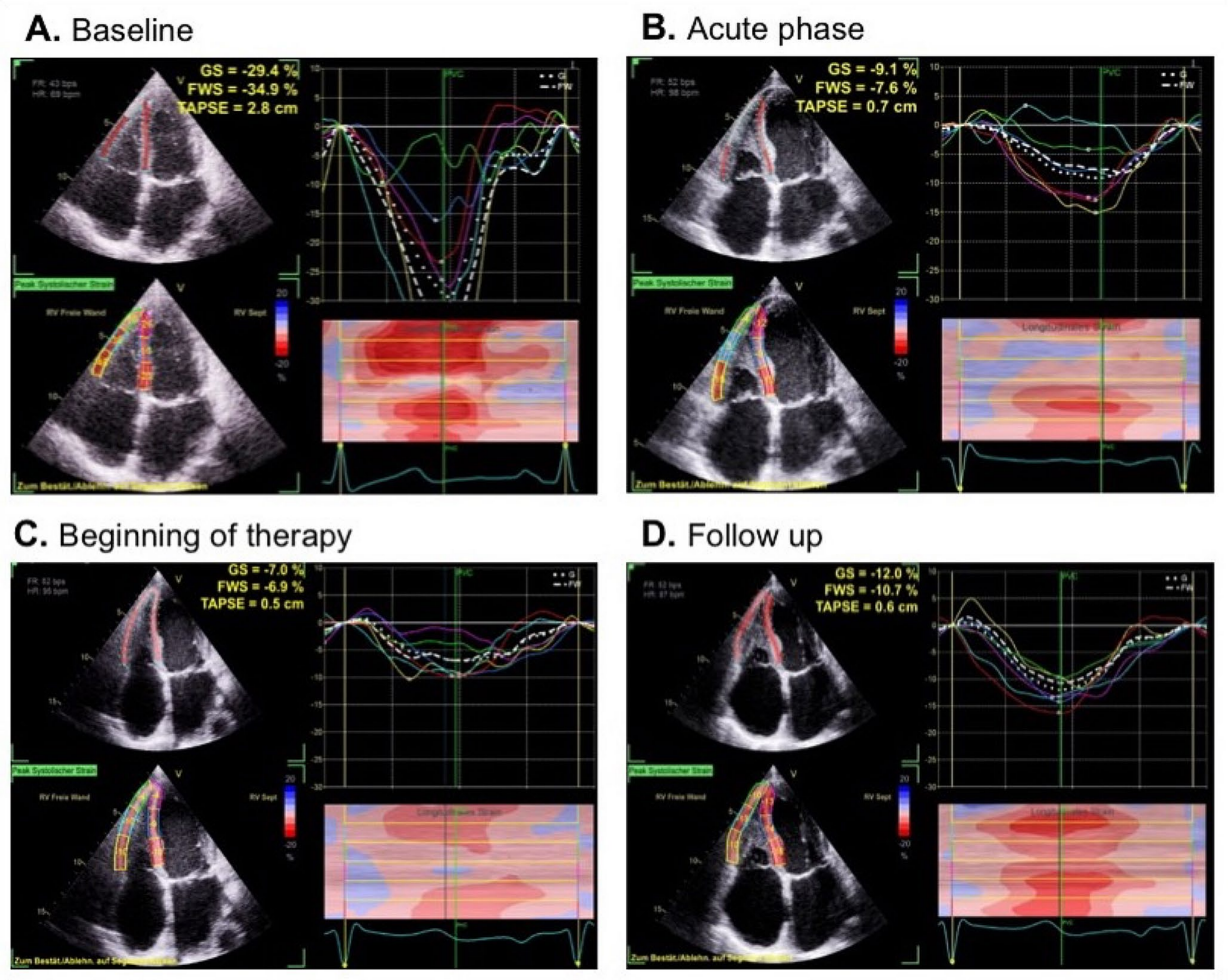
Right ventricular septal and free wall longitudinal strain at baseline (**A**), acute stage of inflammation (**B**), at early prednisolone therapy (**C**) as well as two weeks under ongoing immunosuppressive therapy (**D**). In all illustrations the tracking area (above left), the numerical values of regional peak longitudinal strain (below left), the corresponding regional longitudinal strain graphs (above right) and the corresponding “horse shoe” color coded M-Mode (below right) are presented

**Fig. 5:**
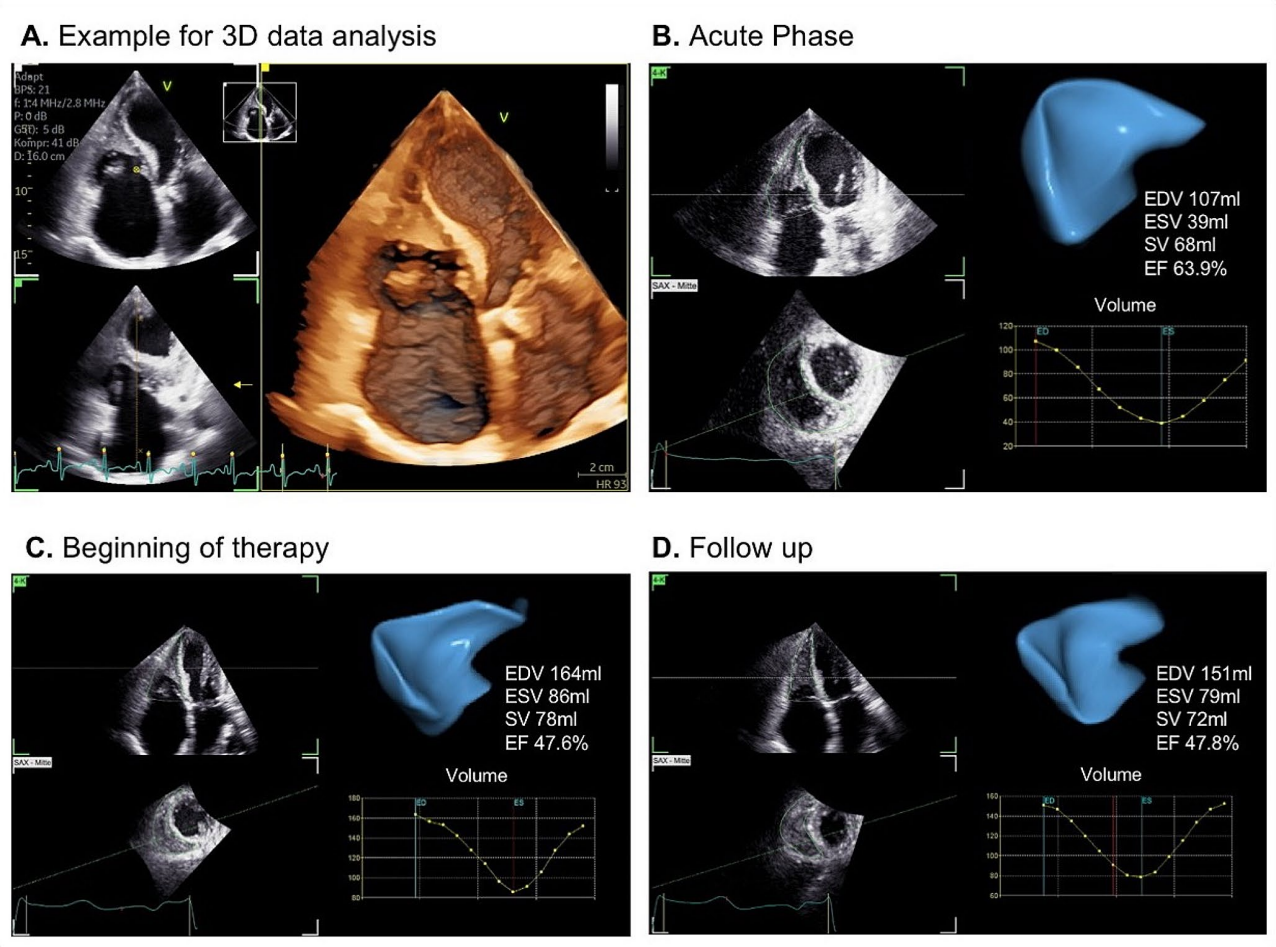
Right ventricular (RV) volume including thrombus formation of the right ventricular apex in a 3D data set at the acute stage of the Loeffler endocarditis (**A**), acute stage of inflammation (**B**), at early prednisolone therapy (**C**) as well as two weeks under ongoing immunosuppressive therapy (**D**). At baseline no 3D data acquisition was performed at routine echocardiography. RV focused 3D data sets were analyzed in the follow-ups

**Fig. 6:**
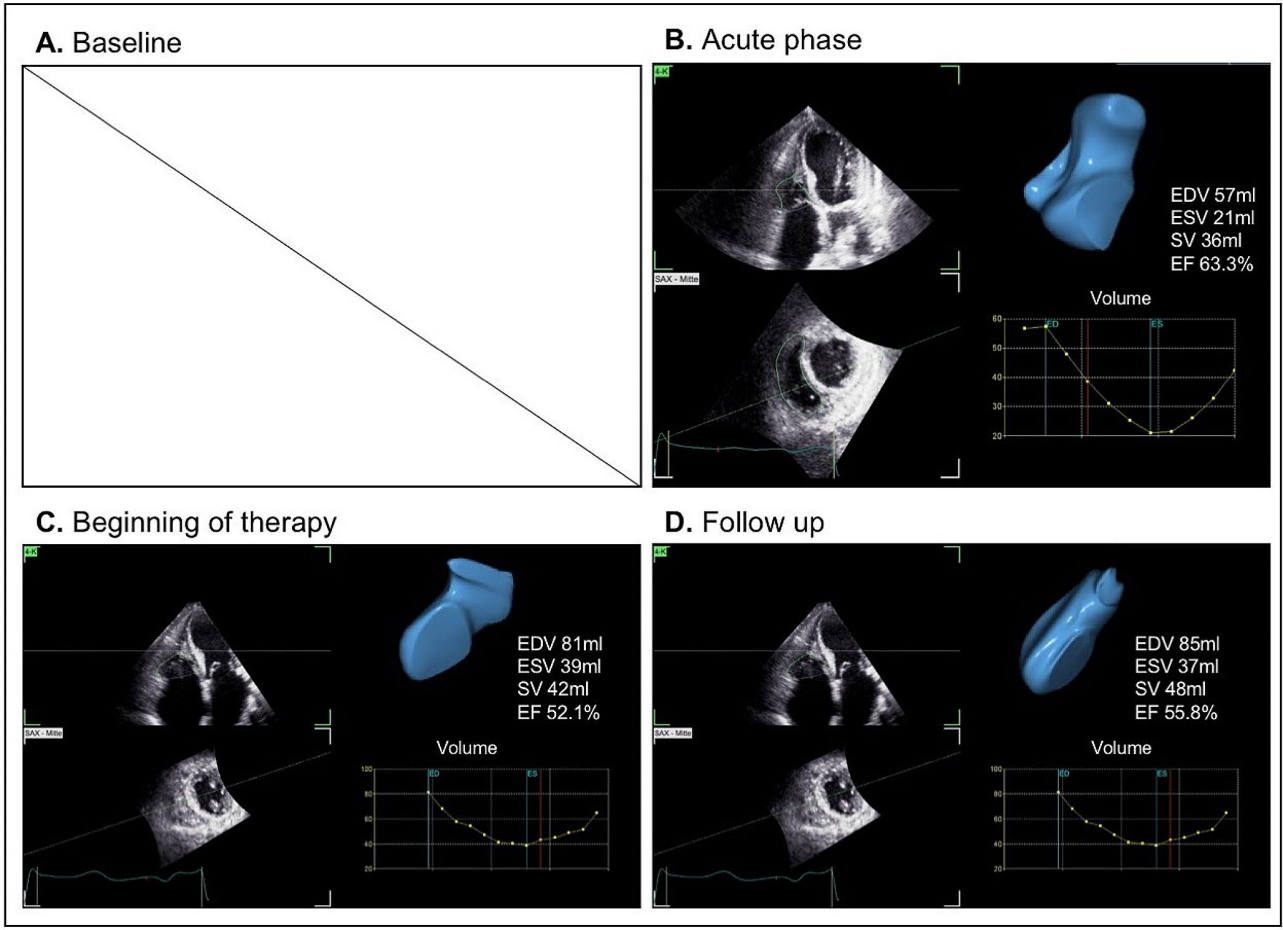
Right ventricular (RV) volume excluding thrombus formation. No 3 data were acquired at baseline (**A**), RV volume analysis at acute stage of inflammation (**B**), at early prednisolone therapy (**C**) as well as two weeks under ongoing immunosuppressive therapy (**D**). At baseline no 3D data acquisition was performed at routine echocardiography (**A**). In the acute phase RV end-diastolic volume (RV-EDV) was 57 ml and RV end-systolic volume (RV-ESV) was 21 ml (**B**). At start of prednisolone therapy RV-EDV was 81 ml and RV-ESV was 39 ml (**C**). During the later follow up RV-EDV was 85 ml and RV-ESV was 37 ml, respectively (**D**). The calculated RV total stroke volumes excluding the RV thrombus formation are 36 ml, 42 ml, and 48 ml, respectively

TTE was firstly performed using the GE Vivid S70 system with the M5Sc probe, while subsequent follow ups were performed using the GE Vivid E95 system with the 4Vc probe. Data analysis was performed with GE EchoPAC software (version 206). LV and RV speckle-tracking echocardiography (STE) of regional longitudinal strain (rLS) was performed by Q-analysis and AFI-RV. 3D-RV volumetry was performed using the 4D Auto RVQ software. The average global longitudinal left ventricular strain (GLS) was calculated by analyzing sectional planes of all three standardized apical views. Patterns of LV rLS were illustrated by bull`s eye plots (Fig. 3). RV global systolic strain (RV GS) and RV free wall systolic strain (RV FWS) were analyzed from RV-focused apical four-chamber views (a4ChV) (Fig. 4). Both parameters represent longitudinal deformation of the combined RV septum and the RV free wall. The RV volume was analyzed by two approaches: Firstly. RV volume was measured by including the thrombotic formation (see Fig. 5) and secondly by excluding the thrombus (see Fig. 6). Thus, total RV stroke volume was determined (RVSV_tot_) cardiac output and cardiac index of the RV and LV were measured by pulsed wave Doppler echocardiography according to current recommendations [14, 15].

At baseline **(**Fig. 4A**)** no systolic motion abnormalities were found in all regions except apical RV free wall. The green tracking area shows the involver apical RV region. However, GS (-29.4%) and FWS (-34.9%) remain in normal range [16] **(**Fig. 4B**)**. Acute phase demonstrates impaired systolic function in the apical region. Global strain value (-9.1%) and FWS (-7.6%) were significantly less negative compared to baseline. At follow up to prednisone therapy **(**Fig. 4C**)** GS was − 7% and FWS was − 6.9%. At follow up **(**Fig. 4D**)** patient`s wall motion improved with GS (-12%) and FWS (− 10.7%). Echocardiographic parameters of LV- and RV-function from baseline to follow up are displayed in Table 1.

**Table 1:** PW Doppler data representation of the left and right ventricular function: effective stroke volume (ESV) calculated from end-diastolic volume and end-systolic volume, cardiac output (CO), and cardiac index (CI) respectively from baseline to follow up

Variable	Baseline	Acute phase	Beginning of therapy	Follow up
Right ventricle				
ESV (ml)	X	36	42	48
CO (L/min)	X	3.3	4.0	4.6
CI (L/min/m^2^)	X	2.1	2.6	2.9
Left ventricle				
ESV (ml)	46	35	34	46
CO (L/min)	3.3	3.2	3.2	4.0
CI (L/min/m^2^)	2.1	3.0	2.1	2.6

## Discussion and conclusion

The present case report focuses on the diagnostic value of echocardiographic monitoring. Speckle tracking echocardiography and 3D echocardiography can detect myocardial involvement due to Loeffler endocarditis at subclinical stages as well as the initial therapeutical effects at early stage. The monitoring of myocardial function by quantitative echocardiography enables an objective approach to visualize relevant cardiac alterations. Thus, these techniques are suitable to detect sudden early deteriorations of cardiac function with the risk of acute heart failure in Loeffler endocarditis, which is described in literature [13, 17–19].

The following learning issues can be made about accompanying echocardiographic imaging:

1. Comprehensive echocardiography can detect subclinical myocardial inflammation prior to the obvious acute disease stage shown by the RV thrombus formation as can be shown by the apical RV strain pathology at baseline.
2. Significant changes of RV and LV involvement due to the inflammatory process can be documented by RV and LV longitudinal strain measurements. Thus, treatment response can be monitored at early stages.
3. Assessment of effective stroke volume by 3D RV volumetry corresponds to LV and RV forward stroke volume determined by Doppler echocardiography. However, thrombus formation of the apical RV cavity must be excluded performing the volumetric analysis.
4. Thus, conventional echocardiography just by visual assessment does not meet the requirements of quantitative monitoring of myocardial function and hemodynamics.

## Electronic supplementary material

Below is the link to the electronic supplementary material.

Supplementary Material 1: Graphical abstract.

## Data Availability

No datasets were generated or analysed during the current study.

## Abbreviations

HES: Hypereosinophilic syndrome
RV: Right ventricular
LV: Left ventricular
CMR: Cardiac magnetic resonance tomography
TTE: Transthoracic echocardiography
STE: Speckle-tracking echocardiography
rLS: Regional longitudinal strain
GLS: Global longitudinal strain
GS: Global systolic strain
FWS: Free wall systolic strain
SV_tot_: Total stroke volume
