# Supplementary figures and images for: Echocardiographic monitoring of myocardial function in a female patient with right heart Loeffler endocarditis at thrombotic stage after Epstein-Barr-virus infection
Source: Int J Cardiovasc Imaging. 2024 May 23;40(9):2007–13. doi: 10.1007/s10554-024-03147-2 (PMC11473627; doi:10.1007/s10554-024-03147-2)

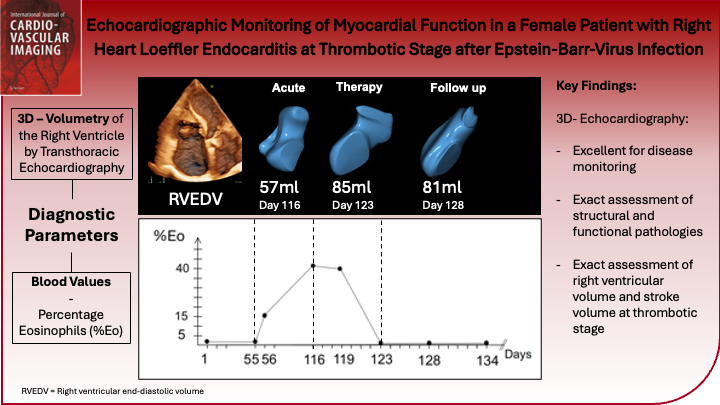

Supplement: Supplementary file 1 — Supplementary Material 1: Graphical abstract. [file 10554_2024_3147_MOESM1_ESM.tiff]
